# Expression and protease characterization of a conserved protein YgjD in *Vibrio harveyi*

**DOI:** 10.7717/peerj.9061

**Published:** 2020-05-18

**Authors:** Yayuan Zhang, Jixiang Chen, Yonggang Wang, Yanlin Li, Wenhong Rui, Jiyi Zhang, Dan Luo

**Affiliations:** 1 School of Petrochemical Engineering, Lanzhou University of Technology, Lanzhou, China; 2 School of life science and enginerring, Lanzhou University of Technology, Lanzhou, China; 3 Chongqing Key Laboratory of Environmental Materials & Remediation Technologies, Chongqing University of Arts and Sciences, Chongqing, China

**Keywords:** Vibrio harveyi, Protease activity, Site-directed mutagenesis, Cell growth

## Abstract

The glycopeptidase GCP and its homologue proteins are conserved and essential for survival of bacteria. The *ygjD* gene (Glycopeptidase homologue) was cloned from* Vibrio harveyi* strain SF-1. The gene consisted of 1,017 bp, which encodes a 338 amino acid polypeptide. The nucleotide sequence similarity of the *ygjD* gene with that of *V. harveyi* FDAARGOS 107 was 95%. The *ygjD* gene also showed similarities of 68%, 67% and 50% with those of *Salmonella enterica*, *Escherichia coli* and *Bacillus cereus*. The *ygjD* gene was expressed in *E. coli* BL21 (DE3) and the recombinant YgjD was purified by Ni^2+^ affinity chromatography column. The purified YgjD showed a specific 37 kDa band on sodium dodecyl sulfate-polyacrylamide gel electrophoresis (SDS-PAGE) and exhibited protease activities of 59,000 units/mg, 53,700 units/mg and 8,100 units/mg, respectively, on N-Acetyl-L-tyrosine ethyl ester monohydrate (ATEE), N-Benzoyl-L-tyrosine ethyl ester (BTEE) and N-Benzoyl-DL-arginine-4-nitroanilide hydrochloride (BAPNA) substrates. When the conserved amino acids of His^111^, Glu^113^ and His^115^ in the YgjD were replaced with alanine, respectively, the protease activities of the mutants were partly decreased. The two conserved His^111^ and His^115^ of YgjD were mutated and the protein lost the protease activity, which implied that the two amino acid played very important roles in maintaining its protease activity. The addition of the purified YgjD to the culture medium of *V. harveyi* strain SF-1 can effectively promote the bacteria growth. These results indicated that the protease activities may be involved in the survival of bacteria.

## Introduction

As one of the top 10 essential proteins (Galperin, 2004), the GCP proteins are described as putative glycoprotease, which were first isolated from *Mannheimia haemolitica*. The GCP and its homologue proteins are conserved in eukaryotes, bacteria and archaea. These proteins were found to be essential for survival both of Gram-positive and Gram-negative bacteria such as *Staphylococcus aureus* (Zheng et al., 2005), *Bacillus subtilis* (Hunt, 2006; Kobayashi et al., 2003), *Mycoplasm genetalium* (Glass et al., 2006), *Francisella novicida* (Gallagher et al., 2007), *Pseudomonas aeruginosa* (Liberati et al., 2006) and *Escherichia coli* (Fabrizio et al., 1998). GCP is also important for the growth of *Saccharomyces cerevisiae* (Downey et al., 2006; Hofmann et al., 2006) and embryo development (Haussuehl et al., 2009) in *Arabidopsis thaliana*.

It has been reported that the GCP homologues are also involved in cell wall biosynthesis, cell division, cell autolysis and antibiotics resistances (Zheng et al., 2007; Bergmiller et al., 2011), and the depletion of protein-coding genes can lead to significant alterations in cell morphology and ultra structure in prokaryotes (Bergmiller et al., 2011; Boyle-Vavra, Yin & Daum, 2006; Nichols et al., 2006). The GCP homolog of *Synechocystis* sp. is also related with its salt tolerance, pigment and cyanophycin production (Karandashova et al., 2002; Zuther, Schubert & Hagemann, 1998). The *ygjD* gene of *E. coli* was previously recognized to consist the *rpsU*-*dnaG*-*rpoD* macromolecular-synthesis operon (Nesin et al., 1987), and involved in the survival by hydrolyzing toxic glycation proteins in *E. coli* (Katz et al., 2010). YgjD has recently been reported to be necessary for the synthesis of universal tRNA N^6^-threonylcarbamoyladenosine (t^6^A) (Hashimoto et al., 2011; Thiaville et al., 2015). YgjD could interact with YjeE and YeaZ to form an YgjD-YeaZ-YjeE complex which determined the function of t^6^A in *E. coli*. It also revealed that the ATPase activity of YjeE is regulated by YgjD-YeaZ heterodimer. Deletion of YgjD, YeaZ or YjeE leads to the loss of the t^6^A biosynthesis (Zhang et al., 2015). The GCP of *S. aureus* is able to regulate the murein hydrolase activities, which further implied the GCP may have multiple function and regulate expression of genes involved in some critical pathways for bacteria survival.

*V. harveyi* is one of the important pathogens of aquatic animals, which poses a serious threat to marine aquaculture (Alvarez, 1998; Wei et al., 2019). Our earlier work revealed that *V. harveyi* could enter into viable but nonculturable (VBNC) state and resuscitated *V. harveyi* cells were found to retain their pathogenicity (Sun et al., 2008). We also found that the purified recombinant YeaZ could improve the resuscitation of the VBNC cells of *V. harveyi* to culturable state (Li et al., 2017). YeaZ also shared some identities with YgjD proteins of *Vibrio* spp. Although the YgjD and YeaZ proteins are inextricably linked, the exact biological functions of the GCP in the bacteria remain unclear. In this study, the *ygjD* gene was cloned and its enzyme activities were evaluated. The promoting effect on cell growth and resuscitation-promoting activities of the purified YgjD were also studied. The underlying mechanisms of resuscitation for bacteria also were explored in this study.

## Material and Methods

### Bacteria strains and plasmids

The main bacteria strains and plasmids are listed in Table 1. The *V. harveyi* strain SF-1 was isolated from diseased sea perch (*Lateolabrax japonicus*) in Qingdao, P. R. China (He, Chen & Li, 2011; Wang et al., 2002), and confirmed by *16S* rDNA sequence analysis (Accession Number: SUB6973071). The *V. harveyi* cells were cultured on Zobell’s 2216 E medium at 30 °C. *E. coil* cells were cultured on Luria-Bertani (LB) medium at 37 °C. All chemical reagents were analytical grade.

**Table 1:** Bacteria strains and plasmids used in this study.

Strains or plasmids	Characteristics	Sources
Strains		
*Vibrio harveyi strain* SF-1	Wild-type	Laboratory collection
*E. coli* DH5 *α*	deoR, recA1, endA1, hsdR17(r_k_^−^, m_k_^+^), phoA, supE44, *λ*^−^, thi^−1^, gyrA96, relA1	Tiangen, China
*E. coli* BL21 (DE3)	*E. coli* B, F^−−^, dcm, ompT, hsdS(r_B_^−^,m_B_^−^), gal, *λ*(DE3)	Tiangen, China
Arctic-Express-pET-28a (+)-*ygjD*	Kan^r^, *ygjD* containing pET-28a (+)	This work
Plasmids		
PEASY-T1 Vector	Kan^r^, Amp^r^, 3.928 kb, high-copy-number cloning vector	TransGen, China
pET-28a (+)	f1 origin; Kan^r^; P_T7_	Zoonbio
PEASY-T1-*ygjD*	Amp^r^, PEASY-T1 with an 1117 bp fragment containing *ygjD* gene of SF-1	This work
pET-28a (+)-*ygjD*	Kan^r^, pET-28a (+) with an 1017 bp fragment containing *ygjD* gene of SF-1	This work

### Cloning and bioinformatics analysis of *ygjD* gene of *V. harveyi*

A pair of primers (forward primer 5′-AATTTTAATCCGATCAAC-3′, reverse primer 5′-TATTTCAAACCTTCAGTAGA-3′) was designed according to the *ygjD* gene of *V. harveyi* and other *Vibrio* spp. on NCBI database (http://blast.ncbi.nlm.nih.gov/). The *ygjD* gene was amplified by PCR from chromosomal DNA of *V. harveyi* strain SF-1. The reaction conditions were as follows: denaturation at 94 °C for 5 min, 30 cycles of denaturation at 94 °C for 60 s, annealing at 42 °C for 1 min, extension at 72 °C for 1.5 min, and a final extension at 72 °C for 10 min. The PCR products were checked by 1.0% agarose gel eletrophoresis and further purified using a DNA purification Kit (Tiangen, Beijing, China). The gene was cloned into PEASY-T1 vector (TransGEN, Beijing, China) and sequenced by Sangon Biotech (Shanghai) Co., Ltd., China. The *ygjD* gene sequence was subjected to similarity alignment analysis on the NCBI. The gene similarities were analyzed with DNAMAN software (version 5.1; Lynnon Biosoft, Quebec, Canada). A phylogenic tree was constructed using MEGA 5.0 by the neighbor joining method with 1,000 replicates.

### Expression and purification of the recombinant YgjD

The *ygjD* gene was amplified by PCR from the PEASY-T1-*ygjD* with a pair of primers (forward primer 5′-GGAGTCTAGAATGCGCATTATTGGTA-3′ with *Xba* I restriction site, reverse primer 5′-CGAATGCGGTTAGCGCGAGCTCCGGC-3′ with *Xho* I restriction site). The PCR products were also detected with the same method above described. The amplified sequence was inserted into pET-28a (+). The recombinant pET-28a (+)-*ygjD* was then transformed into *E. coli* BL21 (DE3). The *E. coli* containing pET-28a (+)-*ygjD* was inoculated into 5 mL of LB liquid medium containing kanamycin (0.05 mg/mL), and cultured overnight at 37 °C with shaking. The culture was then added to 500 mL of the same medium and kept at 37 °C for further 3 h, and then induced with isopropyl-*β*-D-thiogalactopyranoside (IPTG) at 37 °C for 4 h. The bacteria cells were then collected by centrifugation at 10,000 g for 15 min at 4 °C. The cell pellet was resuspended in 20 mL of lysate (pH 8.0, 20 mM Tris-HCl) containing phenylmethanesulfonyl fluoride (PMSF) and protease inhibitor, sonicated (work 4 sec, interval 8 sec, 20 min total) at 400 W by Ultrasonic Processor (SCIENTZ-950E; Ningbo Xinzhi Biotechnology Co., Ltd, Ningbo, China) in an ice bath. The lysate was centrifuged at 9,000 g for 20 min at 4 °C and the precipitate was collected. The inclusion bodies were washed 3 times with inclusion body washing solution (20 mM Tris, 1 mM ethylene diamine tetraacetic acid (EDTA), 2 mM Urea, 1 M NaCl, 1% Triton X-100, pH 8.0), and then were dissolved in a certain ratio with a lysis buffer (20 mM Tris, 5 mM dithiothreitol, 8 mM Urea, pH 8.0). Centrifuged at 15,000 g for 15 min and the inclusion bodies were filtered through a 0.22 µm sterile filter (Millipore, USA). The recombinant protein was purified using a Ni^2+^ affinity chromatography as described (Mukhija & Erni, 1996). The inclusion bodies solutions were loaded into the pre-equilibrium Ni-ID Sepharose CL-6B column, and eluted with Ni-IDA Binding-Buffer at 1.0 mL/min until the absorbance at 280 nm achieved the stable baseline. The targeted protein was eluted with Ni-IDA Elution-Buffer and collected for determining the concentration of protein by Bradford assay (Bradford, 1976). The purified protein was dialyzed with deionized water for analysis by 12% SDS-PAGE (Laemmli, 1970), and protease activity determination.

### Construction, expression and purification of the YgjD mutants

The *ygjD* gene of *V. harvey* i strain SF-1 was analyzed and compared with the other bacteria. The amino acids of the conserved “HXEXH” sequence of the proteases were selected and replaced with other amino acids (Table 2). These mutant gene sequences were synthesized by General Biosystems (Anhui) Co., Ltd., China. The expressing plasmids pET-28a (+) containing mutated *ygjD* gene was constructed and transformed to *E. coli* BL21 (DE3). The recombinant YgjD mutants were expressed by IPTG induction and purified with the abovementioned methods.

**Table 2:** The conserved amino acid residues for substitution and the related oligo nucleotide sequences.

Mutant proteins	Mutations	Oligo nucleotides (5′–3′)*
H111A	His^111^→Ala	CTGTTCACGCTATGGAAGGTCACCTA
E113A	Glu^113^→Ala	CTGTTCACCATATGGCAGGTCACCTACT
H115A	His^115^→Ala	CACCATATGGAAGGTGCCCTACTGGCGCCTAT
H111A+H115A	His^111^→Ala	TTCACGCTATGGAAGGTGCCCTACTGGCGCC
	His^115^→Ala	

### Protein electrophoresis and Western blotting

The purified proteins were detected with western blot method (Das et al., 2018). The purified proteins were cut off acrylamide gels (SDS-PAGE) and transferred on the nitrocellulose membrane after electrophoresis. The membrane was then incubated with rabbit anti-6 ×His tag antibody (Genscript, Nanjing, China) at 37 °C for 2 h. Horseradish peroxidase (HRP)-conjugated goat anti-rabbit immunoglobulin (BoShiDe Biotechnology, Wuhan, China) was used as the secondary antibody. The hybrid membrane was washed with TBST buffer (pH 7.4), and detection was carried out with the ECL Western Blotting Detection kit (SW2010, Solarbio, Beijing).

### Proteolytic activity determination of the purified YgjD

Amidase activity was determined with BAPNA substrate (Erlanger, Kokowsky & Cohen, 1961). Briefly, 0.1 mL of protein solution were mixed with 0.5 mL of BAPNA solution (1 mmol/L), incubated at 37 °C for 30 min, and added 30 µL of 30% acetic acid solution to stop the reaction. The optical densities were detected at a wavelength of 410 nm and the activity was calculated (Erlanger, Kokowsky & Cohen, 1961). 50 µL of protein solution were added into 0.7 mL of ATEE solution (1 mmol/L) or BTEE solution (1 mmol/L), and incubated at 37 °C for 30 min. 30 µL of 30% acetic acid solution were used to stop the reaction. The absorbance at 256 nm were measured and the esterase activities were calculated as described (Hummel, 1959; Schwert & Takenaka, 1955).

### Protease characterization of the recombinant YgjD under different conditions

According to the above described method, the esterase activity of the YgjD also was determined with ATEE substrate at various temperatures (4 °C, 20 °C, 30 °C, 40 °C, 50 °C, 60 °C, 70 °C, 80 °C). The optimal pH and pH stability of the enzyme activities were determined with Na_2_HPO_4_/C_6_H_8_O_7_ buffer (pH 2.0–8.0) and Na_2_HPO_4_/KH_2_PO_4_ buffer (pH 8.0–10.0), respectively. To determine the effect of metal ions on enzyme activities of the recombinant YgjD, different amounts of Mn^2+^, Ca^2+^, Zn^2+^, Co^2+^, Mg^2+^ and Cu^2+^ were added to the mixtures of recombinant YgjD and ATEE solution at 37 °C for 30 min, and the protease activities were determined. Enzyme inhibitors such as DTT, PMSF, EDTA and ethylene glycol tetraacetic acid (EGTA) also were added into the mixtures of recombinant YgjD and ATEE solution at the same conditions, and then dialyzed with deionized water for the enzyme activities determination.

### Effects of the recombinant YgjD proteins on the growth of *V. harveyi* strain SF-1

The *V. harvey* cells were cultured at 30 °C to reach stationary phase, and inoculated into 5 mL of 2216 E broth with addition of the purified wild-type and mutant YgjD proteins, respectively. The bacteria mixtures were then incubated at the same temperature for 72 h. The optical density of bacteria was determined at a wavelength of 600 nm, and the growth curve was drawn for evaluating the effects of the recombinant proteins on the growth of *V. harveyi*.

### Recovery effect of the YgjD on VBNC cells of *V. harveyi* strain SF-1

The *V. harveyi* was cultured in 2216 E broth at 30 °C, 200 rpm overnight. 45 µL of H_2_O_2_ was added to the bacteria solution with a final concentration of 50 mM. The bacteria solution was kept at 30 °C. The viable counts were determined with plate counting method (Kogure, Simidu & Taga, 1979). The bacteria cells were considered to enter into the uncultivable state when the viable cells cannot be detected (Baffone et al., 2006).

5 mL of the VBNC cells were taken out from the VBNC cell microcosm of *V. harveyi*. The cells were collected with centrifugation. The collected cells were washed with sterile physiological saline. They were inoculated into 5 mL of sterile physiological saline. 500 µL of the purified YgjD (0.2 mg/mL) were added to 4.5 mL of the VBNC cells to determine its promoting effect on VBNC cells. A boiled protein solution was used as a control. The mixed solution was kept under the same condition. The culturable cell counts were determined by plate count method.

### Statistical analysis

All the measurements were performed in three separate replicates. Statistical analysis of the mean of the results were performed with SPSS software (SPSS, Chicago, IL). The graphs were constructed using Origin 95 software (OriginLab Corp., USA).

## Results

### Cloning and analysis of *ygjD* gene from *V. harveyi* strain SF-1

**Figure 1:**
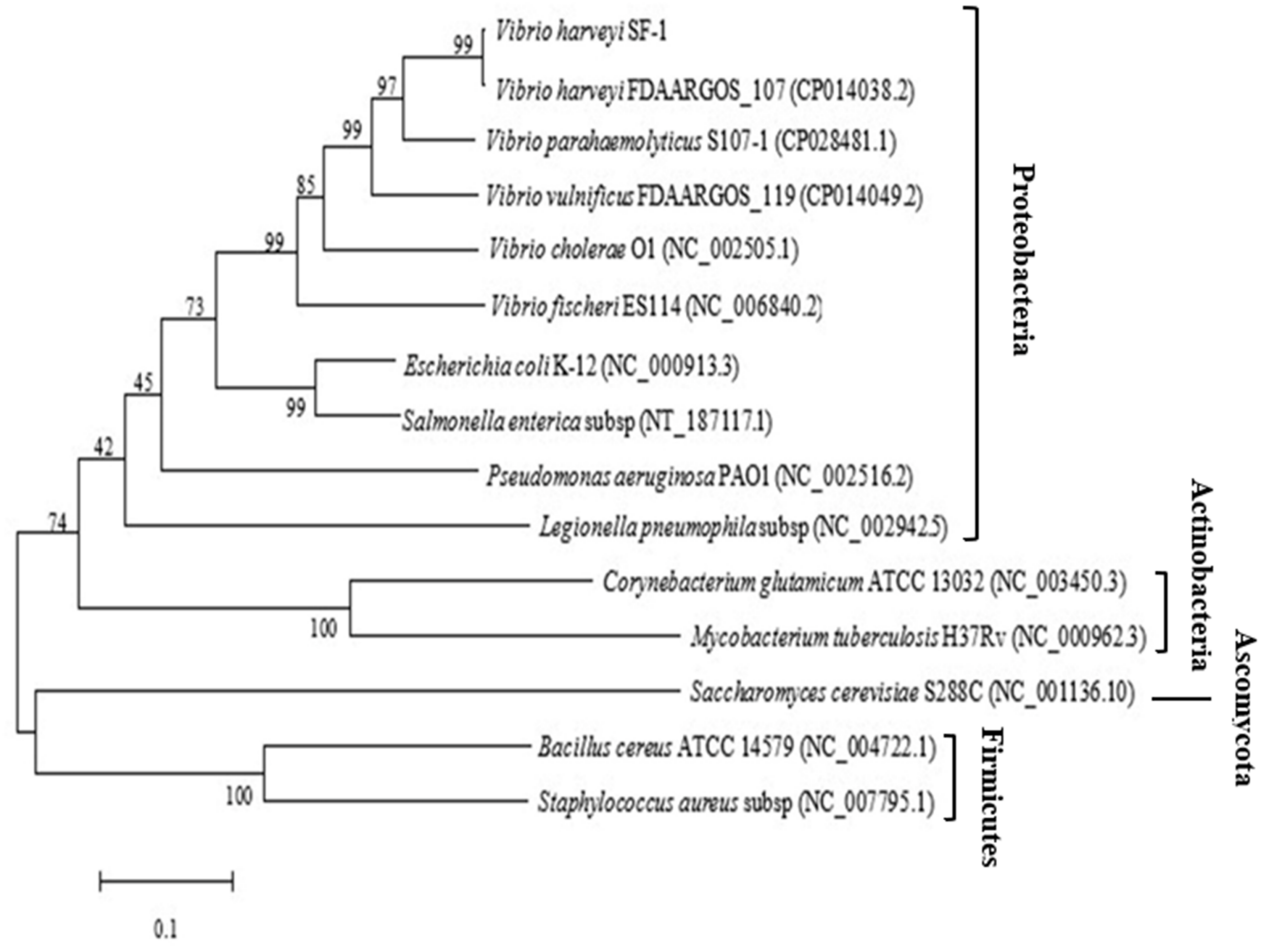
Phylogenetic tree based on the multiple sequence alignment of *ygjD* genes from different strains.

The *ygjD* gene of *V. harveyi* strain SF-1 was composed of 1,017 bp with a stop codon, which encoded a polypeptide of 338 amino acids. The sequence was submitted to NCBI, and an accession was obtained as MN699966. The phylogenic tree from 15 strains were established based on the *ygjD* gene (part segment) as shown in Fig. 1. Its nucleotide sequence showed 95% similarity with that of *V. harveyi* FDAARGOS 107. The nucleotide sequence similarities with *V. cholerae*, *V. vulnificus*, *V. parahaemolyticus* were 75%–83%, respectively. The gene also showed 68%, 67% and 50% similarities with the *ygjDs* of *S. enterica*, *E. coli* and *B. cereus,* respectively. According to the amino acid sequence alignment analysis, the amino acid sequence in this study showed 100% similarity with other *V. harveyi* strains. Meanwhile, the amino acid similarities with *V. cholerae, V. vulnificus* and *V. parahaemolyticus* also reached 89%, 96% and 99%, respectively. However, the similarities with *S. enterica*, *E. coli* and *B. cereus* were 77%, 76% and 43%, respectively. Further it showed 31% similarity with eukaryotic organism *S. cerevisiae*. It was clear from the phylogenetic tree that the cloned *ygjD* gene was more conservative in *Vibrio* sp, and existed in many microorganisms. These results implied *ygjD* gene played an important roles in microbes survival (Zheng et al., 2007; Bergmiller et al., 2011).

### Expression, purification and enzyme activity analysis of the recombinant YgjD

**Figure 2:**
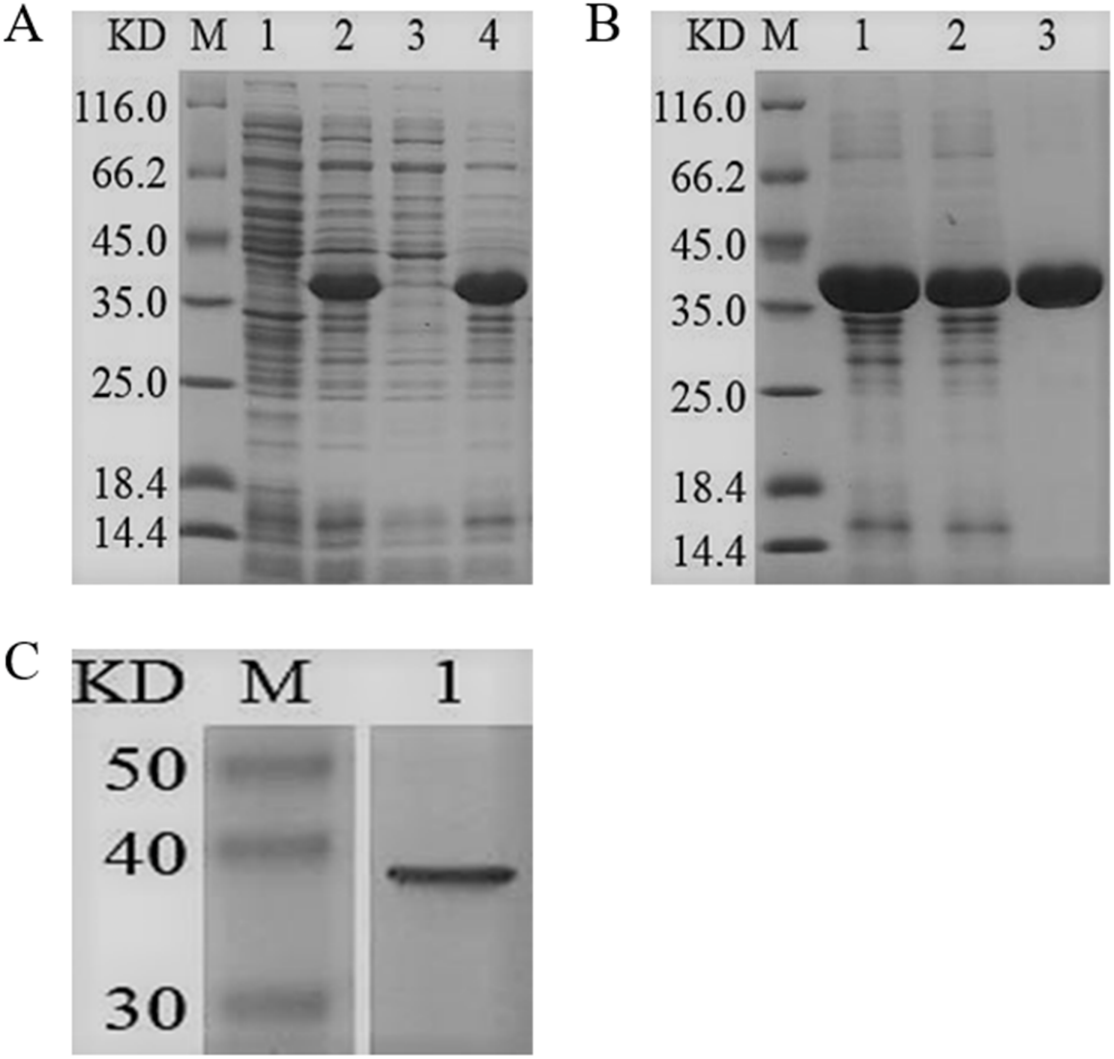
SDS-PAGE and Western blotting analysis of the purified recombinant YgjD. M denotes the middle-ranged protein marker. (A) When the recombination strain was induced for 4 h by IPTG at 37 °C, 20 µL of the culture supernatant was collected. The culture supernatant mixed with the 6× loading buffer were boiled and then determined by SDS-PAGE as shown in band 2 with the un-induced culture supernatant as control (band 1); When the induced cells were cracked by ultrasonic, the supernatant and the precipitation were analyzed by SDS-PAGE (band 3 and 4). (B) The solubilized inclusion body and dialysis solution were measured by SDS-PAGE (band 1 and 2); The purified YgjD by Ni-ID Sepharose CL-6B column was determined shown in band 3. (C) Western blot analysis of the purified YgjD is shown in band 1.

The *ygjD* gene was cloned into pET-28a (+) and the recombined plasmid transformed into *E. coli* BL21 (DE3) for the expression of YgjD protein. The amount of expressed protein purified with Ni^2+^ affinity chromatography column reached at 3 mg. The molecular weight showed a 37 kDa band on SDS-PAGE, which corresponded to the molecular weight of fusion protein (Fig. 2B). The Western blotting also showed a 37 kDa specific band (Fig. 2C), further indicating the protein was correctly expressed and induced by IPTG.

**Figure 3:**
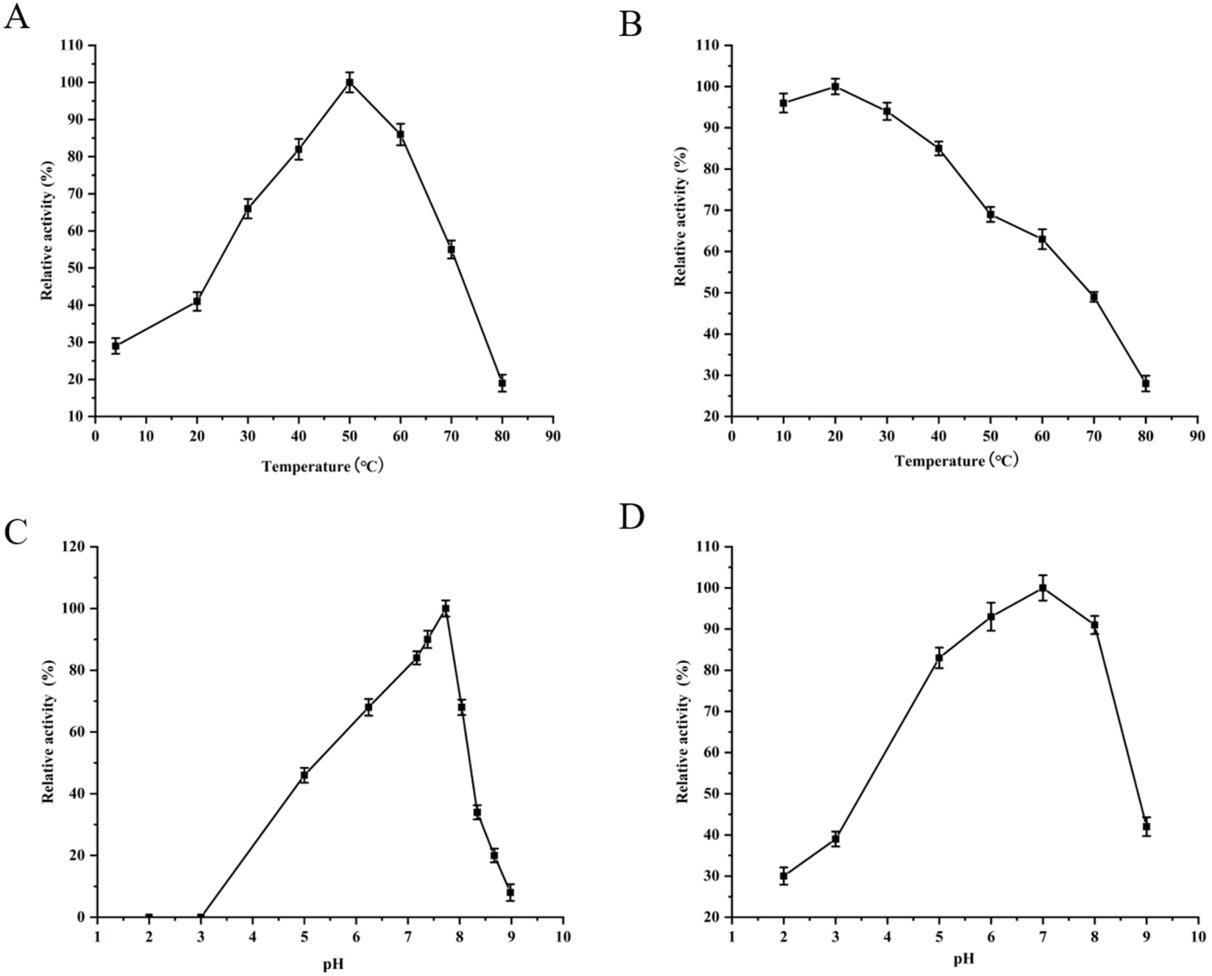
Optimum determination value and effects of temperature and pH on the protease activity of YgjD. (A) Esterase activity of the YgjD with ATEE substrate at various temperatures; (B) esterase activity of the YgjD treated at different temperatures; (C) esterase activity of the YgjD with ATEE substrate at various pH; (D) esterase activity of the YgjD treated at different pH.

The purified YgjD showed protease activities with ATEE, BTEE and BAPNA substrates, the specific activities were of 59,000 units/mg, 53,700 units/mg and 8100 units/mg, respectively. The protein showed maximum activity at 50 °C and pH 7.7 (Fig. 3). As shown in Table 3, Zn^2+^increased the protease activity of the YgjD. Cu^2+^, Mn^2+^, Ca^2+^ and Co^2+^ partly inhibited the enzyme activities. PMSF, EDTA and EGTA all had different degrees of inhibitory effect on the protease activity.

### Mutation analysis of the enzyme active site of the YgjD

**Figure 4:**
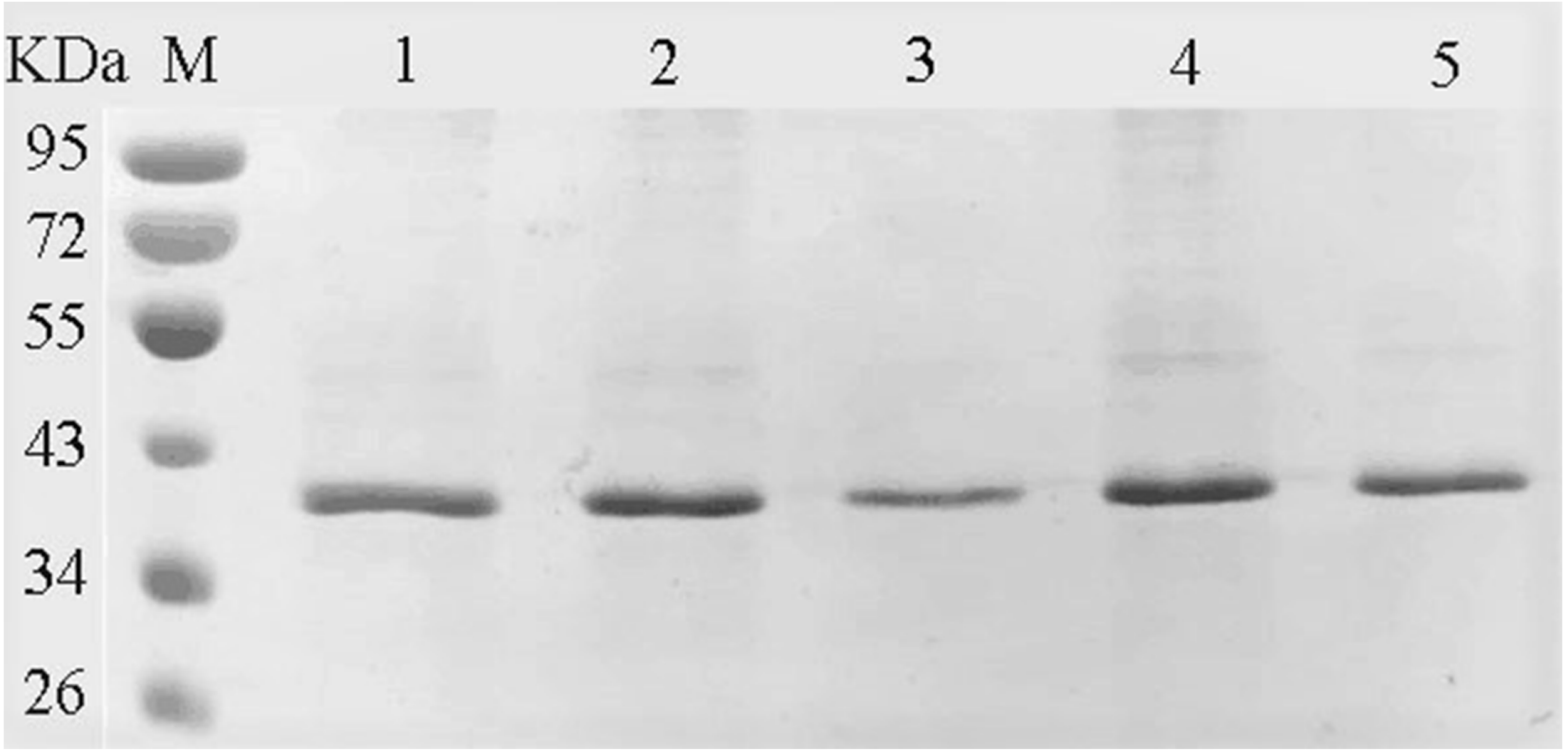
SDS-PAGE analysis of the YgjD mutants and wild-type YgjD. M denotes the middle-ranged protein marker. Bands 1–4 denote the purified YgjD mutants with His^111^, His^115^, His^111^+His^115^ and Glu^113^ by Ni-ID Sepharose CL-6B column, and the wild-type YgjD was used as control (band 5).

The amino acids of the conserved sequence “HXEXH” of the YgjD were separately substituted with alanine. The purified YgjD mutants showed specific bands of the same size with the wild-type protein on SDS-PAGE (Fig. 4). The protease activities of the YgjD mutant were decreased at different degrees (Table 4). When ATEE, BTEE, BAPNA was used as substrate, the protease activities of protein with a single amino acid mutation (H111A, E113A and H115A) decreased significantly. The protease activities with the H111A mutant nearly inactivated with BAPNA substrate, the protease activities with the E113A mutant also inactivated with BTEE substrate, the protease activities with the H115A mutant nearly inactivated with ATEE and BTEE substrates, while the YgjD mutant with His^111^+His^115^(H111A+H115A) lost its activity completely. These results revealed that the two amino acid site (H111A+H115A) played the most important roles in maintaining its protease activity.

**Table 3:** Effects of metal ions and chemical agents on activities of the purified recombinant YgjD of *V. harveyi* strain SF-1. The purified enzyme was pre-incubated with metal ions and chemical reagents for 30 min and dialyzed for activities determination. The enzyme activities of the pre-incubated recombinant protein without reagents were taken as 100%. The activities represent the mean of at least three determinations carried out in duplicates.

Chemical reagents	Concentrations (mM)	Specific activity (u/mg)	Relative activity (%)	Concentrations (mM)	Specific activity (u/mg)	Relative activity (%)
Normal saline	/	4500 ± 200	100.00	/	4580 ± 350	100.00
Zn^2+^	0.1	7020 ± 600	155.55 ± 4.23	1	10850 ± 1100	237.05 ± 101
Mg^2+^	0.1	4580 ± 300	101.85 ± 2.46	1	4440 ± 290	97.05 ± 2.13
Co^2+^	0.1	4220 ± 600	93.82 ± 2.85	1	3520 ± 140	77.06 ± 1.18
Ca^2+^	0.1	3990 ± 150	88.75 ± 3.84	1	3870 ± 230	84.71 ± 2.95
Cu^2+^	0.1	2650 ± 100	59.25 ± 1.34	1	3630 ± 410	79.41 ± 5.23
Mn^2+^	0.1	2330 ± 240	51.85 ± 2.17	1	1670 ± 120	36.47 ± 1.66
EDTA	0.1	3300 ± 320	73.46 ± 1.91	1	2540 ± 370	55.59 ± 3.41
EGTA	0.1	2330 ± 170	51.85 ± 1.85	1	3090 ± 270	67.64 ± 2.04
DTT	0.1	4110 ± 1160	91.36 ± 5.98	1	4440 ± 180	97.05 ± 6.44
PMSF	0.1	2160 ± 260	48.15 ± 1.37	1	1690 ± 160	37.06 ± 0.78

### Effects of the purified YgjD on growth of *V. harveyi* strain SF-1

**Figure 5:**
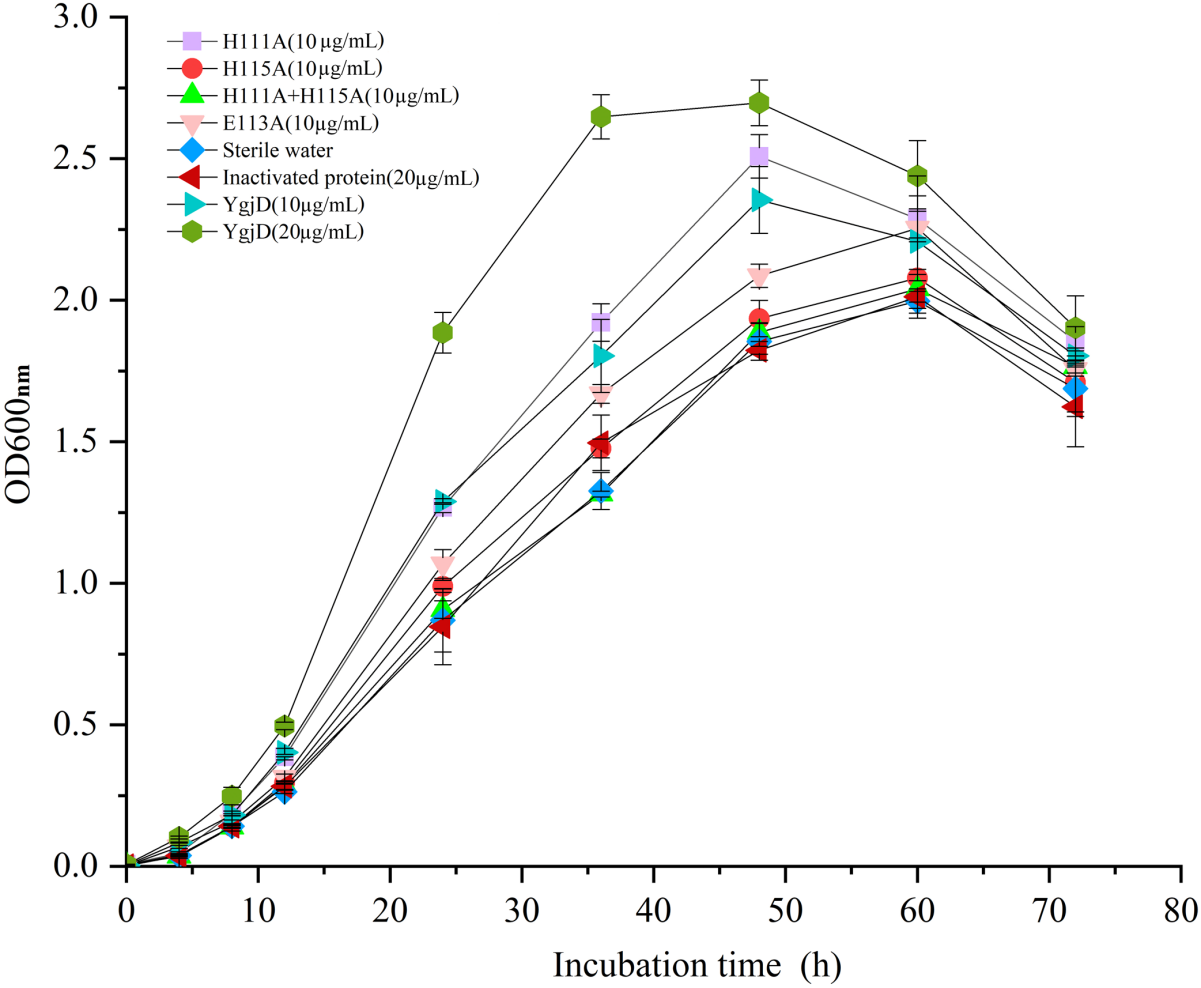
Growth curves of the *V. harveyi* cells in 2216 E broth with addition of the different amount of wild-type YgjD and YgjD mutants.

As shown in Fig. 5, the different growth curve of *V. harveyi* strain SF-1 were determined by addition of the purified YgjD at different concentrations (20 µg/mL and 10 µg/mL), the strain growth rate significantly increased with the concentration increase. And, the cell growth rate increased to 177.01% and 120.52%, respectively, when compared with the control group (20 µg/mL of the boiled YgjD) and normal group (without additive). It’s worth noting that the growth rate of strain SF-1 also increased to 111.56% in the group with the additive of mutant proteins (E113A). However, the growth rate showed no significant difference among mutant proteins (H115A) group, mutant proteins (H111A+ H115A) group, normal group and control group, which implied the YgjD mutant group with His^111^+His^115^ lost its promoting effect on the cell growth. These results further demonstrated that the amino acid (His^111^ and His^115^) played an key function in maintaining the structure and activity of YgjD, and the proteinase activity of YgjD involve in the cell division and cell autolysis (Zheng et al., 2007; Bergmiller et al., 2011).

### Recovery effect of the recombinant YgjD on the VBNC cells of *V. harveyi*

The effects of recombinant YgjD on recovery of *V. harveyi* cells were also analyzed. No visible colonies were observed on the plates added with the purified YgjD protein, which suggested that the YgjD did not have obvious promoting effect on VBNC cells (Table 5).

## Discussion

The YgjD protein was regards as one of GCP homologues, that described as a kind of conserved proteases and their homologues widely exist in bacteria. The GCP belong to MK-M22 O-sialoglycoprotein endopeptidase family. Their amino acid sequences are highly conserved. The two histidines are found at conserved sequence of “HXEXH” and consist of the active centre of the proteases. The two histidines of the “HXEXH” are suggested to coordinate Zn^2+^ and form a catalytic domain (Aravind & Koonin, 1999; Hecker et al., 2007). The GCP isolated from *P. haemolytica* could hydrolyze glycophorin A (Abdullah et al., 1992). Zn^2+^, Cu^2+^, Hg^2+^ and Ni^2+^ inhibited protease activity of the GCP of *P. haemolytica* (Cladman et al., 1996). But the GCP of *S. aureus* did not show any proteolytic activity against glycophorin A. The author explained that the GCP might have some specific proteolytic activities which are critical for the cell viability (Zheng et al., 2005). It has been reported that deletion of *gcp* 1 gene (*ygjD*) in *E. coli* leads to cessation of the cell growth. The GCP1 is related with regulating the cell division. The GCP1 did not show proteolytic activities against *β*-casein, hemoglobin, azocoll and albumin. The author found that GCP1 mutant with the two conserved histidines was not able to rescue the lethal conditional *gcp* 1 mutant phenotype. This result suggested that the two histidines were essential for cell viability of *E. coli* (Lei et al., 2011). The author also found that the N-terminus of the GCP1 is essential for protein to maintain the bacteria viability. Some proteases were reported to hydrolyze low molecular weight peptides and were unable to work on proteins in bacteria (Wiame et al., 2002). Katz reported that the GCP of *E. coli* could metabolize the glycated proteins (Katz et al., 2010). GCP depletion in *E. coli* reduced the growth rate and accumulated active AMPs. The GCP could bind the glycated proteins, elongation factor Tu and pyruvate dehydrogenase (PDH) (Weiß, 2009). Meanwhile, the protease activity of GCP was related to the virulent of microbiome, proteases-mutant strains of GCP was used to live vaccine for vibriosis as described in literatures (Ma, Zhang & Zhao, 2010; Wagner et al., 2013; Mohd-Aris et al., 2019), indicating that GCP proteins have potential applications in microbial growth, resuscitation and vaccine development.

**Table 4:** The enzymatic activities of the wild-type and mutant YgjD with different substrates.

Proteins	Substrates	Specific activity (u/mg)	Percent activity retained (%)
YgjD	BAPNA	8100 ± 400	100.00
	ATEE	59000 ± 5000	100.00
	BTEE	53700 ± 1700	100.00
H111A	BAPNA	0	0
	ATEE	64000 ± 6000	108.39 ± 0.98
	BTEE	28700 ± 3300	53.30 ± 4.46
E113A	BAPNA	6400 ± 600	78.83 ± 3.51
	ATEE	41000 ± 6000	69.12 ± 4.32
	BTEE	0	0
H115A	BAPNA	9000 ± 500	111.07 ± 0.69
	ATEE	0	0
	BTEE	0	0
H111A+H115A	BAPNA	0	0
	ATEE	0	0
	BTEE	0	0

As the abovementioned description, the conserved sequence “HXEXH” of the GCP homologues and the HSP70-actin fold play an important role in the growth and rescues of microorganism. In fact, the same conserved structure also were all found in the YgjD of *V. harveyi*, which showed that the sequence of the *ygjD* gene was highly conserved among bacteria. The *ygjD* gene from *V. harveyi* strain SF-1 consists of 338 amino acids. That showed 100% similarity with other *V. harveyi* strains. It also showed high similarities of 89%–100% with other *Vibrio* species. The similarity with Gram-negative *E. coli* and Gram-positive *B. cereus* was 76% and 43% respectively. Further it showed 31% of the similarity with eukaryotic organism *S. cerevisiae*. The purified protein YgjD did not showed proteolytic activity against azocasein and albumin, but showed the enzymic activity when determined with ATEE, BTEE and BAPNA as substrates. 1.0 mM of Zn^2+^ increased the protease activity of the purified recombinant YgjD, but Cu^2+^, Mn^2+^, Ca^2+^ and Co^2+^ inhibited the enzyme activities to some extent. The amino acids of the conserved sequence “HXEXH” of the YgjD in *V. harveyi* were substituted with alamine. The enzyme activities of the YgjD mutants with His^111^, Glu^113^ and His^115^were greatly decreased, and the YgjD mutant with His^111^+His^115^ lost its proteolytic activity completely. These results showed that the conserved sequence “HXEXH” of YgjD in *V. harveyi* was very important for keeping its protease activity. Some studies have found that the study of *Vibrio* protease is important for understanding its pathogenic mechanism (Liu et al., 2019).

**Table 5:** Effect of wild-type and mutant YgjD on the resuscitation of *V. harveyi* in VBNC state.

	12 h	24 h	48 h	72 h	96 h	120 h
YgjD	0	0	0	0	0	0
Inactivated protein	0	0	0	0	0	0
Sterile water	0	0	0	0	0	0
Sodium pyruvate	0	0.4 ± 0.012	1.7 ± 0.048	2.9 ± 0.036	4.4 ± 0.016	5.1 ± 0.062

**Notes.**

Culturable counts are CFU/mL (×10^3^).

In this work, the resuscitation promoting effect of the recombinant YgjD on the VBNC cells of *V. harveyi* was detected, but no visible colonies were observed on the plates added with the purified recombinant protein. Interestingly, with the addition of the purified YgjD to the normal bacteria culture of *V. harveyi* strain SF-1, the protein could increase the cell growth, and the growth promoting effect was related to the concentrations of the YgjD. But the YgjD mutant with His^111^+His^115^ lost its effects on cell growth, which suggested that the biological function of YgjD might be partly related to the protease activity. The mechanism for its promoting effect remained to be further studied.

## Conclusion

The conserved *ygjD* gene from *V. harveyi* strain SF-1 was cloned and the protein was expressed in *E. coli* BL21. The conserved sequence “HXEXH” of the protease and HSP70-actin fold were found in the YgjD. The purified recombinant YgjD showed protease activities when determined with ATEE, BTEE and BAPNA. Zn^2+^ could increase protease activity, Cu^2+^, Ca^2+^, Mn^2+^, Co^2+^, EDTA, EGTA and PMSF could partly inhibit protease activity. The two conserved His^111^ and His^115^of YgjD played very important roles in maintaining its protease activity. The purified recombinant YgjD protein promoted the normal cell growth, but did not show obvious promoting effect on VBNC cells of *V. harveyi* strain SF-1.

##  Supplemental Information

10.7717/peerj.9061/supp-1 Data S1 Raw data for Fig. 3

10.7717/peerj.9061/supp-2 Data S2 Raw data for Fig. 5

10.7717/peerj.9061/supp-3 Data S3 Raw data for Table 3

10.7717/peerj.9061/supp-4 Data S4 Raw data for Table 4

10.7717/peerj.9061/supp-5 Supplemental Information 5 The full-length blots for Fig. 2C

10.7717/peerj.9061/supp-6 Supplemental Information 6 The data of protease activities using azocasein as substrate

## Additional Information and Declarations

### Data Availability

The following information was supplied regarding data availability: The raw measurements are available in the Supplemental Files.
