# Supplementary figures and images for: Expression and protease characterization of a conserved protein YgjD in Vibrio harveyi
Source: PeerJ. 2020 May 18;8:e9061. doi: 10.7717/peerj.9061 (PMC7241418; doi:10.7717/peerj.9061)

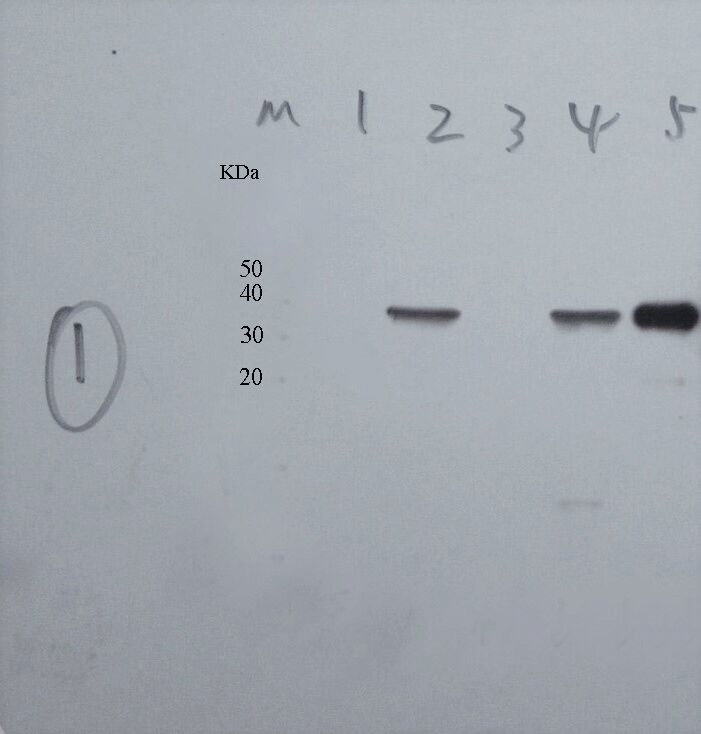

Supplement: Supplemental Information 5 [file peerj-08-9061-s005.zip › WB-YgjD.jpg]
